# Identification, characterization, and utilization of genome-wide simple sequence repeats to identify a QTL for acidity in apple

**DOI:** 10.1186/1471-2164-13-537

**Published:** 2012-10-07

**Authors:** Qiong Zhang, Baiquan Ma, Hui Li, Yuansheng Chang, Yuanyuan Han, Jing Li, Guochao Wei, Shuang Zhao, Muhammad Awais Khan, Ying Zhou, Chao Gu, Xingzhong Zhang, Zhenhai Han, Schuyler S Korban, Shaohua Li, Yuepeng Han

**Affiliations:** 1 Key Laboratory of Plant Germplasm Enhancement and Specialty Agriculture, Wuhan Botanical Garden, the Chinese Academy of Sciences, Wuhan 430074, People’s Republic of China; 2 Graduate School of the Chinese Academy of Science, Beijing 100039, People’s Republic of China; 3 Institute for Horticultural Plants, China Agricultural University, Beijing 100193, People’s Republic of China; 4 Department of Natural Resources and Environmental Sciences, University of Illinois, 1201 W. Gregory, Urbana, IL 61801, USA

## Abstract

**Background:**

Apple is an economically important fruit crop worldwide. Developing a genetic linkage map is a critical step towards mapping and cloning of genes responsible for important horticultural traits in apple. To facilitate linkage map construction, we surveyed and characterized the distribution and frequency of perfect microsatellites in assembled contig sequences of the apple genome.

**Results:**

A total of 28,538 SSRs have been identified in the apple genome, with an overall density of 40.8 SSRs per Mb. Di-nucleotide repeats are the most frequent microsatellites in the apple genome, accounting for 71.9% of all microsatellites. AT/TA repeats are the most frequent in genomic regions, accounting for 38.3% of all the G-SSRs, while AG/GA dimers prevail in transcribed sequences, and account for 59.4% of all EST-SSRs. A total set of 310 SSRs is selected to amplify eight apple genotypes. Of these, 245 (79.0%) are found to be polymorphic among cultivars and wild species tested. AG/GA motifs in genomic regions have detected more alleles and higher PIC values than AT/TA or AC/CA motifs. Moreover, AG/GA repeats are more variable than any other dimers in apple, and should be preferentially selected for studies, such as genetic diversity and linkage map construction. A total of 54 newly developed apple SSRs have been genetically mapped. Interestingly, clustering of markers with distorted segregation is observed on linkage groups 1, 2, 10, 15, and 16. A QTL responsible for malic acid content of apple fruits is detected on linkage group 8, and accounts for ~13.5% of the observed phenotypic variation.

**Conclusions:**

This study demonstrates that di-nucleotide repeats are prevalent in the apple genome and that AT/TA and AG/GA repeats are the most frequent in genomic and transcribed sequences of apple, respectively. All SSR motifs identified in this study as well as those newly mapped SSRs will serve as valuable resources for pursuing apple genetic studies, aiding the apple breeding community in marker-assisted breeding, and for performing comparative genomic studies in Rosaceae.

## Background

The domesticated apple (*Malus* x *domestica* Borkh.) is one of the most economically important tree fruit crops worldwide. The apple is self-incompatible, highly heterozygous, and displays a juvenile period of 6 to 10 years or more. These characteristics render apple breeding programs difficult and time-consuming. To save time and land-space, as well as reduce cost of apple breeding programs, it is imperative to identify young seedlings with desirable traits early and accurately using molecular marker-assisted selection. Hence, identifying molecular markers linked to major genes/quantitative trait loci (QTL) contributing to desirable economic traits is an important goal in apple genetic studies. Several studies have identified QTLs contributing to important horticultural traits, such as resistance to fire blight and to woolly apple aphid [1-4], tree architecture [5,6], and fruit quality components [7].

The cultivated apple is a diploidized autopolyploid species with 17 haploid chromosomes [8]. In recent years, apple genomic resources have greatly expanded, including a large expression sequence tag (EST) database [9,10], a BAC-based genome-wide physical map [11], and a draft sequence of the apple genome [8]. Despite availability of the apple genome sequence, genetic linkage maps remain critical for identification of genomic regions associated with horticultural traits. In apple, several genetic linkage maps have been reported. For example, Maliepaard et al. [12] have developed the first apple linkage map using mostly restriction fragment length polymorphisms (RFLP) and amplified fragment length polymorphism (AFLP) markers. Later, microsatellites or simple sequence repeats (SSRs) have been widely exploited and used to construct high-density linkage maps for apple [13-17]. SSRs, either genomic SSRs (gSSRs) or expressed sequence tag (EST) SSRs (EST-SSRs), are co-dominant, reliable, and highly reproducible. To date, more than 300 gSSRs have been developed and mapped in apple [13-15,17]. More recently, SSRs associated with expressed sequences have also been extensively exploited, and a total of 323 EST-SSRs have been developed and mapped in apple [18]. Despite this progress, the number of SSRs publicly available for apple is not sufficient for the development of high-resolution linkage maps or for rapid saturation of specific map regions, both of which are essential for QTL fine-mapping and positional gene cloning.

Construction of a genetic map requires analysis of hundreds of markers over a relatively large number of plants. Thus, genotyping analysis is a labor-intensive and time-consuming undertaking. During the past several years, rapid progress has been made in developing molecular tools to enable large-scale segregation analysis in genetic studies. PCR-based markers adapted to large-scale genotyping systems can be designed for constructing genetic linkage maps. SSRs are amenable for analysis using automated DNA sequencers, and thus can be adapted for high-throughput genotyping. For example, fluorescent microsatellite genotyping has been successfully carried out recently to develop a high-density linkage map for apple within a few months [18].

In addition to their usefulness in constructing linkage mapping, SSRs are useful for population genetic studies as well as for comparative genomics efforts [19]. Genome-wide analysis of SSRs is not only an efficient strategy to develop abundant molecular markers, but may also provide insights into possible roles of SSRs in chromosome structure, function, and evolution [20]. Therefore, it is important to continue to develop SSR markers for further progress of genetics and genomics efforts for apple. To date, there are a few reports on genome-wide characterization of microsatellite sequences in the apple genome [13,14]. Recently, the apple genome has been sequenced (database is available at http://www.rosaceae.org/projects/apple_genome), thus providing an opportunity to identify and develop robust genome-wide SSRs. In this study, distribution and variation of size of microsatellites within the DNA sequence of the apple genome have been characterized. The aim of this study is to develop SSRs for constructing genetic linkage maps to identify QTLs for fruit acidity. Our results will aid in conducting apple genetic studies, pursuing efficient apple breeding, and performing comparative genomic studies in rosaceae.

## Results

### Simple sequence repeats in the apple genome

The distribution of microsatellites of minimum lengths of 20 bp in assembled contig sequences of the apple genome was analyzed [8]. A total of 28,538 microsatellites, consisting of a variety of repeat types, were identified (Table 1). Di-nucleotide repeats were the most abundant, accounting for 71.9% of all SSRs. Tri-, tetra-, penta-, and hexa-nucleotide repeats accounted for 12.3%, 6.3%, 0.9%, and 0.2%, respectively, of all SSRs. Of the di-nucleotide repeats, AT/TA was the most abundant, accounting for 32.8% of all di-nucleotide repeats, while AG/GA and AC/CA repeats accounted for 30.7% and 8.5%, respectively. It is worth noting that SSR motifs represented variants of both strands of the DNA sequence. GC/CG repeats were rather rare, and only a single CG repeat was found. Among tri-nucleotide repeats, AAC/ACA/CAA was the most abundant, accounting for 39.8% of all tri-nucleotide repeats, followed by AAT/ATA/TAA (22.0%) and AAG/AGA/GAA (22.0%). Of tetra-nucleotide repeats, AAAT/TAAA/ATAA/AATA was the most abundant, accounting for 35.8% of all tetra-nucleotide repeats, and followed by TACA/ACAT/CATA/ATAC (28.1%). Among penta- and hexa-nucleotide repeats, AT-rich repeats were the most abundant, accounting for 24.5% and 11.6% of all penta- and hexa-nucleotide repeats, respectively. Moreover, 63.5%, 88.4%, 91.5%, 98.5%, and 93.4% of di-, tri-, tetra-, penta-, and hexa-nucleotide repeats, respectively, were less than 30 bp in length. A small number of di-nucleotide repeats (15.0%) were longer than 50 bp in length, whereas few tri-, tetra-, pentra-, or hexa-nucleotide repeats (< 5%) were longer than 40 bp in length. Briefly, AT/TA and AG/GA di-nucleotide repeats were the most abundant SSRs in the apple genome, and most SSR motifs were shorter than 30 bp in length.

**Table 1:** Composition and length distribution of major SSR types in the apple genome

**Repeat unit**	**Repeat type**	**Repeat length (bp)**				**Total**	**Frequency (%)**
		**<30**	**30-40**	**40-50**	**>50**		
**Dimer**	AC/CA	1883	357	106	73	2419	8.48
	AG/GA	5115	2090	953	595	8753	30.67
	AT/TA	6035	1968	854	495	9352	32.77
	CG	1	0	0	0	1	0.00
	Total	13033	4415	1913	1163	20524	71.92
**Trimer**	AAT/ATA/TAA	591	97	41	44	773	2.71
	AAC/ACA/CAA	1342	44	7	3	1396	4.89
	AAG/AGA/GAA	643	66	20	44	773	2.71
	Others	520	31	4	6	561	1.97
	Total	3096	238	72	97	3503	12.27
**Tetramer**	AAAT/TAAA/ATAA/AATA	631	10	0	1	642	2.25
	AATT/ATTA/TTAA/TAAT	93	0	0	0	93	0.33
	AAAG/AAGA/AGAA/GAAA	109	10	2	2	123	0.43
	TACA/ACAT/CATA/ATAC	404	76	15	8	503	1.76
	GTTT/TGTT/TTGT/TTTG	90	1	1	0	92	0.32
	AGGG/GAGG/GGAG/GGGA	91	4	0	0	95	0.33
	Others	222	17	5	1	245	0.86
	Total	1640	118	23	12	1793	6.28
**Pentamer**	AAAAT/AAATA/AATAA/ATAAA/TAAAA	494	2	0	0	496	1.74
	GTTTT/TGTTT/TTGTT/TTTGT/TTTTG	154	1	0	0	155	0.54
	AATTT/ATTTA/TTTAA/TTAAT/TAATT	94	1	0	0	95	0.33
	AAAAG/AAAGA/AAGAA/AGAAA/GAAAA	115	0	0	0	115	0.40
	AAGCC/AGCCA/GCCAA/CCAAG/CAAGC	170	0	0	0	170	0.60
	CCCTG/CCTGC/CTGCC/TGCCC/GCCCT	121	7	0	1	129	0.45
	Others	747	15	1	1	764	2.68
	Total	1895	26	1	2	1924	6.74
**Hexamer**	AAAAAT/AAAATA/AAATAA/AATAAA/ATAAAA/TAAAAA	106	2	2	0	110	0.39
	AAAAAG/AAAAGA/AAAGAA/AAGAAA/AGAAAA/GAAAAA	38	1	0	0	39	0.14
	AAAAAC/AAAACA/AAACAA/AACAAA/ACAAAA/CAAAAA	34	0	0	0	34	0.12
	CCCTCT/CCTCTC/CTCTCC/TCTCCC/CTCCCT/TCCCTC	33	2	2	0	37	0.13
	ATACAT/TACATA/ACATAT/CATATA/ATATAC/TATACA	40	6	0	1	47	0.16
	AGAGTG/GAGTGA/AGTGAG/GTGAGA/TGAGAG/GAGAGT	21	1	0	0	22	0.08
	CCTCTC/CTCTCC/TCTCCC/CTCCCT/TCCCTC/CCCTCT	35	3	1	0	39	0.14
	Others	434	23	7	1	465	1.63
	Total	741	38	12	2	793	2.78

SSRs with at least 20 bp in length and more than 3 repeats were recorded. SSR motifs represent variants of both strands of the DNA sequence (e.g., AC/CA includes the reverse complements GT and TG).

**Figure 1:**
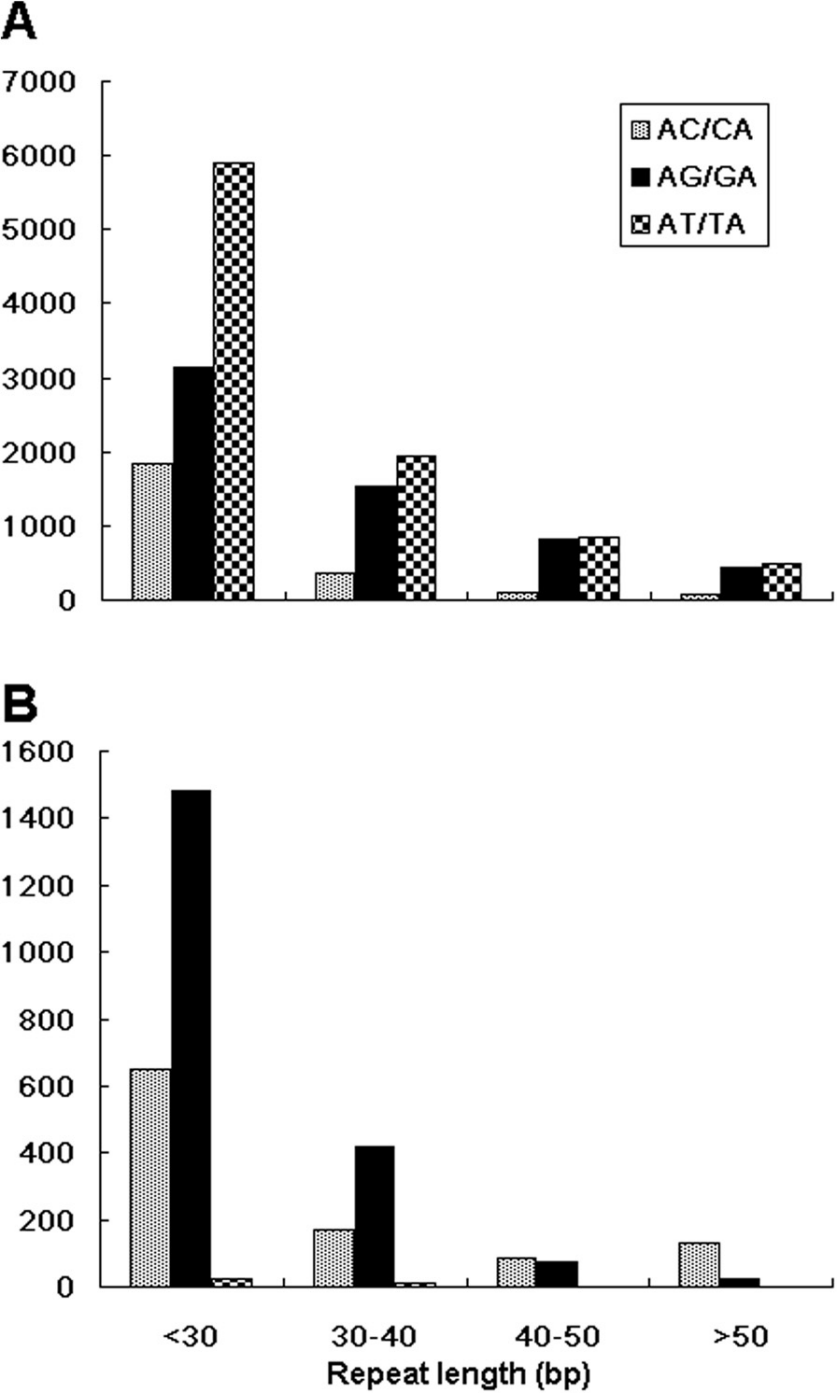
Distribution of dimeric SSRs in genomic DNA (A) and transcribed sequences (B) of the apple genome

To investigate the distribution of SSRs in coding DNA sequences (CDSs), untranslated regions (UTRs), and genomic regions of the apple genome, DNA sequences flanking SSR motifs were compared with both the apple CDS database (http://www.rosaceae.org/projects/apple_genome) and expression sequence tag (EST) database of NCBI. Of 28,538 SSRs in scaffold sequences of the apple genome, 513 (1.8%), 4,120 (14.4%), and 23,905 (83.8%) were present in CDSs, UTRs, and genomic DNA, respectively (Table 2). Of CDS-SSRs, tri-nucleotide repeats were the most abundant, accounting for 51.7% of all CDS-SSRs, and followed by hexa-nucleotide repeats (28.5%). In contrast, among UTR-SSRs or gSSRs, di-nucleotide repeats were the most abundant, accounting for 73.0% and 72.9% of all UTR-SSRs and gSSRs, respectively. AG/GA dimers prevailed in transcribed sequences and accounted for 66.8% of all UTR-SSRs. Of all gSSRs, AT/TA and AG/GA dimers accounted for 38.3% and 24.8%, respectively. Briefly, AT/TA and AG/GA were the most abundant in genomic DNA and transcribed sequences of the apple genome, respectively (Figure 1).

**Table 2:** Distribution of SSR types in coding DNA sequences (CDSs), untranslated regions (UTRs), and genomic DNA of the apple genome

**Repeat**	**Region**	**Repeat length (bp)**				**Total**	**Frequency (%)**
		**<30**	**30-40**	**40-50**	**>50**		
Dimer	CDS	76	0	0	0	76	0.27
	UTR	2098	598	158	153	3007	10.54
	Genomic	10860	3817	1755	1010	17442	61.12
Trimer	CDS	260	5	0	0	265	0.93
	UTR	425	22	3	2	452	1.58
	Genomic	2411	211	69	95	2786	9.76
Tetramer	CDS	10	0	0	0	10	0.04
	UTR	158	11	1	1	171	0.60
	Genomic	1472	107	22	11	1612	5.65
Pentamer	CDS	16	0	0	0	16	0.06
	UTR	223	7	0	0	230	0.81
	Genomic	1658	19	1	2	1680	5.89
Hexamer	CDS	136	8	1	1	146	0.51
	UTR	248	10	2	0	260	0.91
	Genomic	355	20	9	1	385	1.35

### SSR polymorphisms in apple cultivars and wild species

As demonstrated above, most SSR motifs in the apple genome were either di- or tri- nucleotide repeats. Thus, 310 primer pairs flanking 278 di-nucleotides repeats and 32 tri-nucleotide repeats were designed to test polymorphism among eight apple genotypes, including four cultivars and four wild species (Table 3). Of 310 primer pairs, 298 (96.1%) were amplified, and 245 (79.0%) showed polymorphic banding patterns in all genotypes tested. The average numbers of alleles amplified by gSSRs, UTR-SSRs, and CDS-SSRs were 3.5, 2.4, and 2.6, respectively. The average number of alleles amplified by each SSR was 3.2 for the four apple cultivars and 3.4 for the four wild apple species; moreover, each dimer or trimer detected 3.4 and 2.5 alleles, respectively, in these two sets of genotypes. The average PIC values for gSSRs, UTR-SSRs, and CDS-SSRs were 0.64, 0.48, and 0.54, respectively, for all genotypes tested. The average PIC value for all SSR loci was 0.57 for four apple cultivars, and with a higher average PIC value of 0.66 for the four wild apple species. On average, gSSRs flanking AC/CA, AG/GA, and AT/TA motifs had 2.8, 3.5, and 3.2 alleles in all eight genotypes, respectively. The average PIC values of AC/CA, AG/GA, and AT/TA motifs in genomic regions were 0.52, 0.67, and 0.63, respectively, over all eight genotypes.

**Table 3:** Average allele numbers and PIC values of SSRs in eight apple genotypes

**SSR region**	**Type**	**No. of SSRs**	**Average allele number**			**Average PIC value**		
			C*	W*	C+W*	C*	W*	C+W*
CDS	Dimer	3	2.50	2.75	2.63	0.44	0.48	0.46
	Trimer	9	2.53	2.71	2.62	0.55	0.59	0.57
UTR	Dimer	28	2.26	2.56	2.41	0.49	0.51	0.50
	Trimer	11	2.27	2.27	2.27	0.41	0.45	0.43
Genomic	Dimer							
	AC/CA/TG/GT	17	2.40	3.20	2.80	0.44	0.60	0.52
	AG/GA/TC/CT	102	3.20	3.69	3.45	0.62	0.73	0.67
	AT/TA	63	2.75	3.19	2.97	0.56	0.69	0.63
	Total	182	3.51	3.67	3.59	0.60	0.71	0.65
	Trimer	12	2.57	2.57	2.57	0.52	0.56	0.54

C: Four apple cultivars, including ‘Golden Delicious’, ‘Jonathan’, ‘Luao’, and ‘Starkrimson’. W: Four wild apple species, including *M. prunifolia* (Willd.) Borkh. (‘Regunzihaitang’), *M. sieversii* (Lebed.) Roem. (‘Xinjiangyepingguo 12’), *M. × robusta* Rehd. (‘Pingding Crab’), and *M. asiatica* Nakai (‘Naizi’). C+W: Both four apple cultivars and four wild apple species.

Among all 310 primer pairs, 81 primer pairs were identified to be polymorphic between ‘Golden Delicious’ and ‘Jonathan’, parents of the F_1_ mapping population. Thus, these primer pairs were used to construct a genetic linkage map for apple, and these were designated with a ‘WBGCAS’ prefix to distinguish them from previously published SSRs. Primer sequences of these newly identified SSRs are listed in Additional file 1.

### Construction of a genetic linkage map of apple

**Figure 2:**
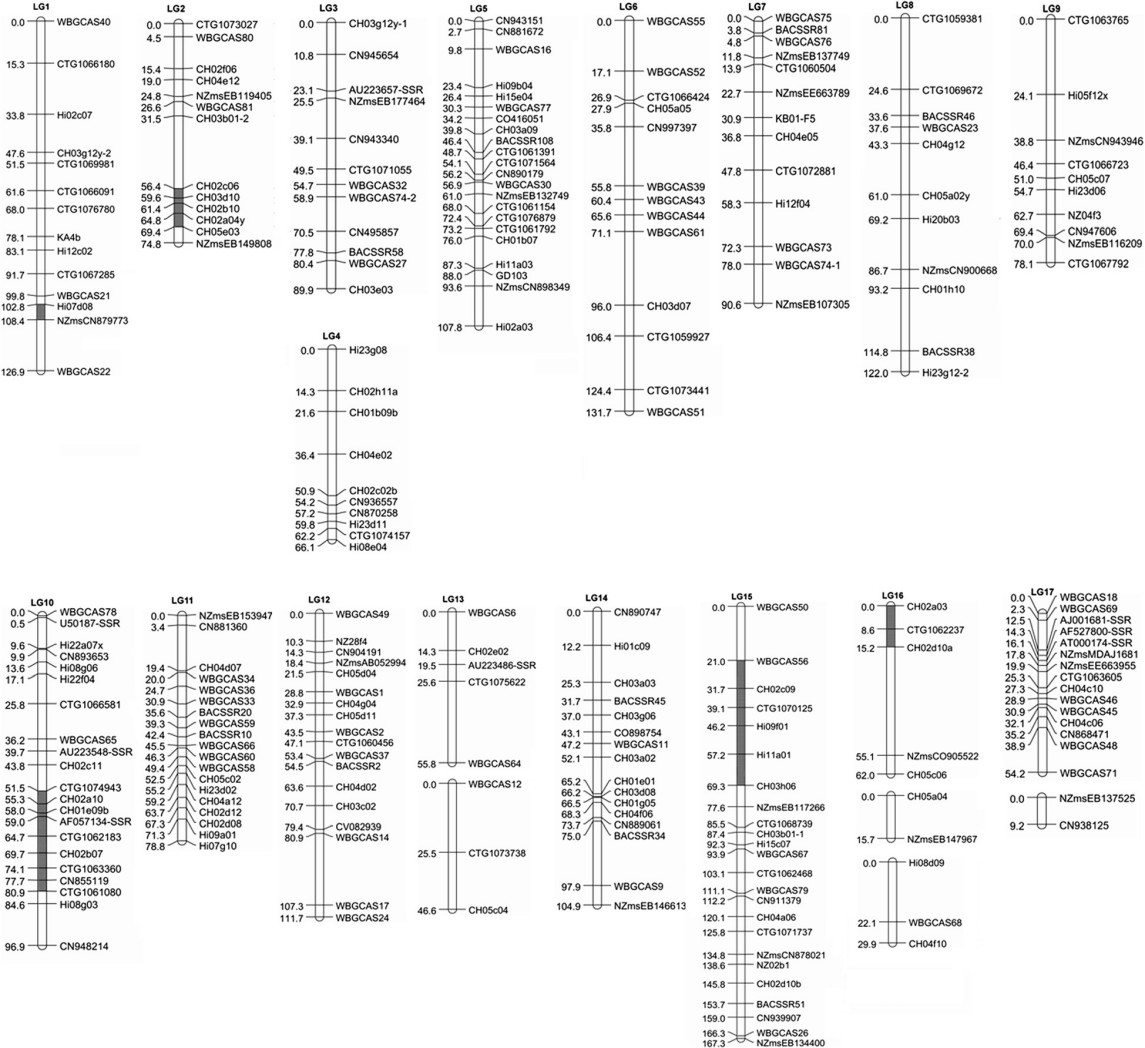
**SSR-based genetic map of apple.** M: male parent (cv. ‘Golden Delicious’), F: female parent (cv. ‘Jonathan’), and LG: linkage map. The consensus map is shown in centre, and distances shown in the maps are measured in centimorgans (cM). The number of linkage groups corresponds to the number of the haploid chromosomes of the draft apple genome sequence [8]. Segregation-distorted markers clustered within the chromosomal region are indicated in gray.

A total of 676 previously published SSR markers were initially used to screen the parents ‘Golden Delicious’ and ‘Jonathan’. Of 676 SSR markers, 327 were selected from a public domain of apple molecular markers (http://www.hidras.unimi.it/) and 349 were recently developed EST-SSRs and BAC-end sequence (BES)-SSRs [18,21]. As a result, 218 SSRs (98 gSSRs, 15 BES-SSRs, and 105 EST-SSRs) were identified to be polymorphic between ‘Golden Delicious’ and ‘Jonathan’. These polymorphic SSRs, together with the newly-developed SSRs with a ‘WBGCAS’ prefix designation, as described above, were then used to screen all seedlings of the mapping population of ‘Jonathan’ x ‘Golden Delicious’. This revealed presence of five segregation types, as defined in JoinMap 4.0, including lm×ll, nn×np, hk×hk, ab×cd, and ef×eg (Additional file 2). Of 299 tested SSRs, 12 amplified two loci. As a result, a total of 311 loci were scored. Subsequently, 50 loci were excluded from linkage analysis as they either failed to link with any of the linkage groups or their distorted segregation conflicted with segregation patterns of neighboring markers. Moreover, 44 seedlings were found to carry several double recombination events, and were excluded from further linkage analysis. Finally, a consensus linkage map consisting of 251 loci (91 l m × ll, 95 nn × np, 15 hk × hk, 15 ab × cd, and 50 ef × eg) along 21 linkage groups was successfully generated (Figure 2). These 21 linkage groups were assigned to their respective chromosomes, based on previously published linkage maps [12-18]. Each linkage group had 8 to 24 markers with an average of 14.7. The consensus linkage map spanned 1720.9 cM with an average density of 6.8 cM per marker.

Of 251 SSR loci along the consensus map, 141 and 148 were mapped onto linkage maps of ‘Jonathan’ and ‘Golden Delicious’, respectively. The total lengths of linkage maps of ‘Jonathan’ and ‘Golden Delicious’ were 1228.4 and 1403.9 cM, respectively, and the average densities were 8.7 and 9.4 cM per SSR, respectively.

### Identification of QTLs responsible for malic acid content in ripe apple fruit

Of 242 F1 seedlings used to construct the linkage map, 162 were reproductive and bore fruits. Collected fruits from these seedlings were evaluated for malic acid contents. The average content of malic acid for this segment of the mapping population was 2.42 mg/g, ranging from 0.44 to 6.58. Interval mapping was conducted for malic acid content, and a QTL for malic acid content was detected on linkage group 8 of ‘Jonathan’, and this was flanked by markers CH04g12 and Hi20b03. The QTL explained ~ 13.5% of the phenotypic variation, with an LOD score of 3.4. In the Kruskal–Wallis analysis, the *Ma* QTL was supported with a highly significant (P < 0.0001) value of the *K* statistic, 15.9 for malic acid content.

## Discussion

### Frequency and variation of SSRs identified in the apple genome

Currently, there is no consensus on the definition of SSRs, particularly regarding the minimum length of repeat sequences [22]. In this study, the distribution and frequency of SSRs has been analyzed, with repeat unit lengths of 2 to 6 bp and a minimum length of 20 bp in assembled contigs of the DNA sequence of the apple genome. A total of 28,538 SSRs were identified in the apple genome (Additional file 3). Given the estimated 700 Mb size of the apple genome [8], the SSR density is ~ 40.8 per Mb in the DNA sequence of apple. This observed SSR frequency in the apple genome is lower than those reported for other plant species. For example, overall densities of SSRs in genomes of *Arabidopsis*, rice, and cucumber are 874, 807, and 551 SSRs/Mb, respectively [23,24]. This is probably mainly due to the fact that higher stringent conditions have been used in defining SSRs in this study than those used previously for *Arabidopsis*, rice, and cucumber. For *Arabidopsis*, rice, and cucumber, di- and tri-nucleotide repeats of minimum lengths of 12 di-nucleotide repeats have been recorded, along with tetra- to hexa-nucleotide repeats of at least three repeat units [24]. Moreover, it is worth mentioning that the total amount of repetitive elements in apple is estimated to be 352.6 Mb in size, representing ~ 47.5% of the whole genome sequence [8]. Whereas, repetitive elements in *Arabidopsis*, cucumber, and rice account for 18.5%, 14.8%, and 39.5% of their whole genome sequences, respectively [8]. It has been reported that microsatellites are preferentially associated with non-repetitive DNA in plant genomes [25]. Thus, abundance of repetitive sequences in the apple genome may also contribute to the observed low frequency of SSRs.

More recently, Cavagnaro et al. [24] have analyzed microsatellites in different plants, and found that tri-nucleotide repeats are the most prevalent type of SSRs in *Arabidopsis*, soybean, rice, and sorghum, while tetra-nucleotide repeats are most prevalent in poplar and grapevine. In this study, di-nucleotide repeats are the most abundant SSRs in apple, accounting for ~ 71.9% of all SSRs. This clearly suggests that repeat units of the most abundant SSRs in different plants vary considerably in size. However, it is not known whether or not this variation is related to plant speciation. Moreover, most SSRs identified in apple are less than 30 bp in length, and very few SSRs are longer than 50 bp. The observed distribution of lengths of SSRs in this study is consistent with previous findings that the frequency of repeat types decreases exponentially with repeat lengths [26].

In apple, SSRs are strongly biased towards AT-rich repeat motifs. For example, AT/TA repeats are not only the predominant dimers, but they are also the most frequent motifs in the entire genome. Moreover, only a single GC repeat with at least 20 bp in length is present in the apple genome. Similarly, among all tri-, tetra-, penta-, and hexa-nucleotide repeats found in the apple genome, AT-rich repeats are the most abundant SSRs. However, GC-rich motifs are quite few, and only 7, 2, and 9 of GGC/CCG/GCG/CGC, GCCG/GGGC, and CCCCG/GCCCG/GCGGG repeats, respectively, are detected in the apple genome. These results are in agreement with previous findings that AT-rich SSRs are predominant in such dicots as *Arabidopsis*[27], papaya [28], soybean [29], and cucumber [24]; while GC-rich repeats are predominant in monocots, and most are present in coding regions [30]. It has been reported that GC contents in monocots are generally higher than those found in dicots [24]. Thus, the basal composition of the genome may play an important role in determining the types of observed SSRs in higher plants.

The predominance of tri- and hexa-nucleotide repeats in coding DNA sequences has been widely reported in several plant species [23,24,31]. In this study, tri- and hexa-nucleotide repeats prevail in coding DNA sequences of apple, accounting for 51.7% and 28.5% of all CDS-SSRs, respectively. The abundance of tri- and hexa-nucleotide repeats in plants may be attributed to negative selection against frame-shift mutations. Moreover, CCG/CGC/GCC repeats prevail in coding DNA sequences of rice [23]; while AGG/GAG/GGA repeats are the most abundant in coding DNA sequences of apple. Thus, positive selection for specific single amino-acid stretches may be involved in expansion of tri-nucleotide repeats in plants [25].

In this study, gSSRs detected more alleles and higher PIC values than either CDS-SSRs or UTR-SSRs. This is consistent with previous findings that suggested that EST-SSRs have lower levels of allele variations than gSSRs [22]. Di-nucleotide repeats prevail in apple, and the most frequent motifs in genomic and transcribed regions are AT/TA and AG/GA, respectively. Of dimeric gSSRs, AG/GA motifs have revealed more alleles and higher PIC values than AT/TA or AC/CA motifs. AG/GA repeats in transcribed sequences have detected higher PIC values in eight apple genotypes than in other dimeric EST-SSRs. In a previous study, 825 EST-SSRs used to evaluate polymorphisms in two apple genotypes, ‘Co-op 16’ and ‘Co-op 17’, have shown that 28.8%, 22.1%, and 15.8% of AG/GA, AT/TA, and AC/CA repeats are polymorphic, respectively [18]. Therefore, it seems that AG/GA repeats in either genomic or transcribed regions may be more variable when compared with their counterparts of AT/TA and AC/CA repeats in apple. The AG/GA repeats may be more efficient than any other types of SSRs for genetic diversity studies and for linkage map construction in apple. In addition, SSRs have detected more alleles and higher PIC values in the four apple cultivars than in the four wild apple species. This suggests that SSR motifs in apple are less variable in cultivars than in wild species, which may be attributed to domestication of the apple.

### Transferability and segregation distortion of apple SSRs among populations

Recently, a total of 312 EST-SSRs were mapped onto an apple linkage map using the mapping population ‘Co-op 17’ × ‘Co-op16’ [18]. In this study, these EST-SSRs were selected to construct a genetic linkage map, and 64 (20.5%) were found to be polymorphic between ‘Jonathan’ and ‘Golden Delicious’. Moreover, of 296 previously reported gSSRs, prefixed with ‘CH’ or ‘Hi’ [17,32,33], used to screen the two parents ‘Jonathan’ and ‘Golden Delicious’, 94 (31.8%) gSSRs were found to be polymorphic between these two parents and 90 (30.4%) gSSRs were successfully anchored onto the genetic linkage map. Similarly, of 254 previously published gSSRs, prefixed with ‘CH’ or ‘Hi’ [17,32,33], used to construct a linkage map for ‘Co-op 17’ × ‘Co-op16’, 81 (31.9%) displayed polymorphisms between ‘Co-op 17’ and ‘Co-op 16’ [18]. This indicated that the level of transferability of apple SSRs was not high among populations, and gSSRs exhibited higher levels of polymorphism than EST-SSRs (~ 32% versus ~ 21%).

The above findings may be attributed to the fact that DNA sequences are known to be conserved in expressed regions. Moreover, among 310 gSSRs identified from the assembled genome sequence of apple selected to amplify both apple cultivars and wild species, 48.3% are found to be polymorphic among cultivars and wild species; whereas, only 21.6% are polymorphic between ‘Jonathan’ and ‘Golden Delicious’. Therefore, using the mapping population from a cross between a domesticated cultivar and a wild species would aid in constructing an SSR-based genetic linkage map for apple.

In addition to low levels of transferability observed in this study, a high frequency of segregation distortion (20.7%) was also observed among apple SSRs. Segregation distortions have been reported in other apple mapping populations, and up to 27.5% of markers have shown segregation distortion in a cross between ‘Wijcik McIntosh’ and ‘NY75441-58’ [12,16,32,34]. A high frequency of segregation distortion (20.3%) has also been noted for SSRs in grapevine [35]. Segregation distortion is generally reported to be due to presence of lethal genes influencing viability of gametes and/or zygotes [36]. Therefore, a cluster of markers within the chromosomal region surrounding lethal genes will always show segregation distortion [37]. In apple, clustering of markers with distorted segregation has been previously observed for linkage group 10 [12,32,34]. In this study, 5, 9, 6, and 3 SSRs with distorted segregations are clustered within a region on consensus linkage groups 2 (56.4 – 69.4 cM), 10 (51.5 – 80.9 cM), 15 (21.0 – 69.3 cM), and 16 (0.0 – 15.2 cM), respectively (Figure 2). These segregation distortion regions may contain genes influencing viability of gametes and/or zygotes [32]. Moreover, two SSRs, Hi07d08 and NZmsCN879773, with distorted segregation are also found to be clustered on linkage group 1 (Figure 2). When comparing linkage group 1 in this study with previously reported genetic linkage maps, it is found that these two SSRs are linked to *Vf* genes for scab resistance in apple [18,38]. It has been reported that *Vf* genes in apple are linked to sub-lethal genes [39]. Thus, such lethal genes are likely to be responsible for the observed distorted segregation of the two clustered SSRs Hi07d08 and NZmsCN879773. In addition, SSRs with distorted segregation across the same linkage group are also observed in this study. For example, three SSRs, including CTG1066180, ctg1076780, and Hi12c02 show distorted segregation, and are located in different regions of linkage group 1. Thus, it seems that other factors such as chromosome loss and self-incompatibility may be also involved in segregation distortion in apple [40,41].

### Utilization of SSR motifs identified from assembled genome sequences of apple

Initially, almost all published SSRs were used to construct the genetic linkage map for apple using F_1_ seedlings from the cross between ‘Jonathan’ and ‘Golden Delicious’. However, only ~ 200 SSRs were found to be polymorphic between these two parents. To aid in subsequent linkage map construction, developing additional SSR markers was deemed necessary. Therefore, SSR motifs in assembled genome sequences of apple were analyzed, and more than 300 SSRs across the whole genome were selected to screen the two parents of the mapping population used in this study. As a result, 81 additional gSSRs were developed, and an SSR-based linkage map of apple was successfully developed. These results demonstrated that exploring and using SSRs from the draft of the apple genome sequence were efficient for constructing a genetic linkage map. As more than 28,000 SSR motifs were present in the apple genome, this allowed for developing an SSR-based high-density linkage map using a high-throughput genotyping technology such as the fluorescent capillary electrophoresis [18].

In this study, a total of 54 gSSRs, identified from the apple genome sequence, were genetically mapped onto 15 linkage groups. Mapping results of these gSSRs were compared with their positions along the draft of the apple genome sequence [8]. Surprisingly, 13 out of 54 gSSRs revealed inconsistencies between their genetic-map positions and sequence-based physical-map positions (Additional file 4). Similarly, when comparing DNA sequences flanking SSRs against the apple genome sequence, 23 previously developed SSRs showed discrepancies between their genetic-map and sequence-based physical-map positions (Additional file 4). Moreover, primer sequences of 36 discrepant SSRs were also compared against the apple genome sequence, and results indicated that all these SSRs were likely to be single loci. Additionally, genetic-map positions of 196 previously developed SSRs on the consensus linkage map were compared with earlier results. Of 196 SSRs, 192 were mapped onto the same linkage groups as previously reported [17,18,21,32,33]. Two SSRs, CH01b09b and CH04g12, were for the first time genetically mapped onto linkage groups 4 and 8, respectively. Previously, the two gSSRs CH03b01 and Hi08g03 were mapped onto linkage groups 2 and 6, respectively [15,17]; however, in this study, CH03b01 was mapped onto two linkage groups 2 and 15, while Hi08g03 was mapped onto linkage group 10. CH03b01 and Hi08g03 have been reported to be multi-locus SSRs [15,17], and linkage group 2 was homologous to linkage group 15 [8,18]. Thus, the genetic mapping results of CH03b01 and Hi08g03 observed in this study are likely to be accurate.

Overall, the genetic positions of published SSRs in this genetic linkage map are consistent with previous reports, thus suggesting that mapping results of SSRs from the draft sequence of the apple genome are reliable. However, it seems that a small portion of assembled contig sequences of the apple genome may not be correctly anchored onto apple chromosomes. Of 36 discrepant SSRs, nine are located on linkage group 10 (Additional file 4), indicating that the draft sequence of chromosome 10 may be less reliable when compared with those of other chromosomes.

### QTLs for fruit acidity

Acidity plays an important role in determining fruit quality, and several studies have been carried out to identify QTLs responsible for fruit acidity in both apple and peach [12,16,32,42]. In apple, a major QTL or *Ma* gene, responsible for fruit acidity, has been mapped onto linkage group 16, and this can explain ~ 30% of the observed variance [32,43]. In addition to the *Ma* gene, six other QTLs for apple fruit acidity have also been detected on linkage groups 2, 8, 10, 13, 15, and 17 [32,43]. In this study, the newly developed linkage map of apple has been used to identify QTLs for fruit acidity. As malic acid is the major acid in apples [44], malic acid content instead of fruit pH or titratable acidity has been used to characterize apple fruit acidity in this study. A QTL responsible for malic acid content of apple fruits is detected on linkage group 8 of cv. ‘Jonathan’. This QTL is linked to SSR marker CH05a02y, and this is consistent with the finding reported by Liebhard et al. [45]. Thus, it seems that the QTL for apple fruit acidity detected in this study is reliable, although the QTL analysis has been performed using phenotyping data from a single year (2009), as most seedlings have not fruited either due to incidence of damaging cold temperatures during flowering or outbreak of canker disease. It is worth noting that the effect of QTLs for apple fruit acidity may have been underestimated due to low marker density of linkage map groups and the small mapping population size. Additionally, some linkage groups are split into two with gaps of unknown lengths. The large gaps in some linkage groups, together with low marker density as well as the small population size may all have contributed to inability in detecting some QTLs with small effects. In this study, a QTL with a peak LOD value of 2.2 is detected on linkage group 5, but this QTL is not recorded as its LOD value is lower than the cutoff threshold value of 2.8.

## Conclusions

This study provides insights into the characteristics of microsatellites in apple. Overall the apple genome, di-nucleotide repeats are the most frequent SSRs, accounting for 71.9% of all SSRs. A key new finding is that among these di-nucleotide repeats, AT/TA and AG/GA are the most frequent in genomic and transcribed regions of apple, respectively. AG/GA repeats are more variable than AT/TA or AC/CA repeats in apple. A total of 310 primer pairs of SSRs have been designed to assess their polymorphisms, and 245 (79.0%) are found to be polymorphic in eight apple genotypes. The newly developed SSRs in this study, together with previously published SSRs, have been used to construct a genetic linkage map for apple using an F_1_ population derived from a cross between ‘Jonathan’ and ‘Golden Delicious’. The genetic mapping results indicate that gSSRs have higher levels of polymorphism among different mapping populations of apple than EST-SSRs. Distortion-segregated markers have been clustered along several chromosome regions. A QTL responsible for malic acid content of apple fruits has been detected on linkage group 8 of apple cv. ‘Jonathan’.

Briefly, the apple genome is rich in di-nucleotide repeats, and AG/GA repeats are more variable than other dimers. The availability of a very large set of microsatellite markers distributed throughout the genome may facilitate a variety of genomic studies in apple, including development of high-resolution linkage maps, positional gene-cloning, and fine-mapping of QTLs/genes.

## Methods

### Plant material

A segregating F_1_ population derived from a cross between ‘Jonathan’ and ‘Golden Delicious’, maintained at the Changli Institute of Pomology (Hebei Province, PRC), was used for linkage map construction. The segregating population consisted of 286 individual seedlings. Young leaves of these apple seedlings and their parents were collected for DNA isolation.

### Identification of SSRs

The assembled sequence of the ‘Golden Delicious’ apple genome was downloaded from the Genome Database for Rosaceae (http://www.rosaceae.org/projects/apple_genome). Assembly sequences of the apple genome were scanned for perfect microsatellites (uninterrupted run of repeats) using the computer program MIcroSAtellite identification tool (MISA). SSRs recorded for the final dataset included dimers to hexamers of at least 20 bp in length.

### Analysis of SSR genotyping

Primers were designed based on flanking sequences of SSRs using the Primer 3 program (http://primer3.sourceforge.net/). Amplification was performed under the following conditions: 3 min at 95°C, followed by 35 cycles of 45 s at 94°C, 45 s at 55°C, 45 s at 72°C, and a final extension step at 72°C for 10 min. Five μL of amplification products was mixed with an equal volume of formamide loading buffer (98% formamide, 10 mM EDTA, pH 8.0, 0.025% bromophenol blue and xylene cyanol). The mixture was denatured at 94°C for 3 min, and then immediately chilled on ice. An aliquot of 2 μL mixture was loaded on a 6% polyacrylamide gel, and electrophoresed for 1.5 h at 1200V. Bands were visualized after silver staining, and recorded on a ScanMaker 3830 (Microtek, Shanghai, China).

### Investigation of polymorphism of SSRs in apple cultivars and wild species

Evaluation of SSR polymorphism was conducted using four apple cultivars, including ‘Golden Delicious’, ‘Jonathan’, ‘Luao’, and ‘Starkrimson’, along with four wild apple species, including *M. prunifolia* (Willd.) Borkh. (‘Regunzihaitang’), *M. sieversii* (Lebed.) Roem. (‘Xinjiangyepingguo 12’), *M. × robusta* Rehd. (‘Pingding Crab’), and *M. asiatica* Nakai (‘Naizi’). SSRs exhibiting polymorphisms among the eight apple genotypes were subjected to calculated polymorphism information content (PIC) values following the protocol described by Zhang et al. [46].

### Construction of a genetic linkage map and QTL analysis

A total of 286 F_1_ seedlings derived from a cross between ‘Jonathan’ and ‘Golden Delicious’ were used for the construction of genetic linkage maps for apple. The linkage analysis was carried out using JoinMap version 4.0 [47], as previously described by Han et al. [18]. Briefly, an LOD score threshold of 8.0 was initially used to assign markers to different linkage groups. Once linkage groups were determined, the remaining markers were added to their corresponding groups using a less stringent criterion of LOD score of 4.0. Genetic maps for each parent were constructed using the function of ‘Create Maternal and Paternal Node’ in the JoinMap program. The regression mapping algorithm was used for map construction. Map distances were calculated using Kosambi’s mapping function, and denoted in centiMorgans (cM). Once the genetic maps for each parent were constructed, a consensus map was built using the CP population model. Order of markers in genetic maps for each parent was used as preferred orders (the ‘fixed order’ function) for the construction of a consensus map.

QTL analysis was conducted using MapQTL v4 [48]. Genetic linkage maps for each parent were used to detect QTLs using an interval mapping approach, and an LOD score of 2.8 was set as a genome-wide threshold to declare significant QTLs.

### Analysis of malic acid content in apple fruits

Three apple fruits from each progeny were collected at maturity, cut into small sections, and then stored at −70°C until use. One gram of fresh fruit was ground into a fine power in liquid nitrogen, and dissolved in 10 mL of 80% methanol (pH 7, 0.1M imidazole). The mixture was incubated for 15 min at 75°C, sonicated for 20 min in an ultrasonic water bath, and centrifuged at 10,000 g for 10 min at room temperature. A total of 980 μL of the supernatant and 20 μL of an inner standard (2.5% methl-α-D- glucopyranoside, 2.5% phenyl-β-D-glucopyranoside, and 10% acetone) were mixed, and then centrifuged at 10,000 g for 20 min at room temperature. An aliquot of 0.5 mL from each sample was evaporated to dryness, and the residue was dissolved in 0.8 mL of hydroxylamine hydrochloride solution in pyridine (20 mg/ml). The mixture was incubated at 75°C for 1 h, and then cooled down to room temperature. Subsequently, 0.4 mL of hexamethyldisilazane and 0.2 mL of trimethylchlorosilane were added. Following incubation at 75°C for 2 h, the mixture was centrifuged at 12,000 g for 20 min at room temperature. A total of 0.5 mL of supernatant was subjected to gas chromatography analysis (Agilnet 6890N, USA) to estimate the concentration of organic acid content as described by Morvai and Molnár-Per [49].

## Competing interests

The authors declare that there are no competing interests.

## Authors’ contributions

QZ, BM, HL, YC, and YYH participated in SSR genotyping and linkage analysis. MAK, SSK, and SL participated in QTL mapping and manuscript preparation. XZ and ZH developed the segregating populations. JL, SZ and GW collected phenotype data. YPH was overall project leader. All authors read and approved the final manuscript.

## Supplementary Material

Additional file 1 Primer sequences of newly developed SSRs in apple. Click here for file

Additional file 2 **Five segregation types of SSRs in the F**_1_ mapping population of ‘Jonathan’ x ‘Golden Delicious’. Click here for file

Additional file 3 Details of SSRs, their primer sequences, and sequences of SSR motifs and their flanking regions in the apple genome. Click here for file

Additional file 4 **Apple SSRs revealing discrepancies between genetic-map and sequence-based physical-map positions.** A: Linkage maps of this study, B: Linkage map of previous studies [17,18,31,32], C: The apple draft map. SSRs were anchored onto the draft map of apple by comparing DNA sequences flanking SSRs against the apple genome sequences of cv. ‘Golden Delicious’. Click here for file
