# Supplementary material for: Identification, characterization, and utilization of genome-wide simple sequence repeats to identify a QTL for acidity in apple
Source: BMC Genomics. 2012 Oct 7;13:537. doi: 10.1186/1471-2164-13-537 (PMC3704940; doi:10.1186/1471-2164-13-537)
Supplement: Additional file 1 — Primer sequences of newly developed SSRs in apple. [file 1471-2164-13-537-S1.doc]

**Additional File 1: Primer sequences of newly developed SSRs in apple**

| **Name** | **Primer** | | **Tm (℃)** |
| --- | --- | --- | --- |
|  | **Forward** | **Reverse** |  |
| WBGCAS1/2 | TCAACCAACGACAAATCAAGGG | GCCCACGAGTTCGAGGAATTTG | 55.5 |
| WBGCAS3 | GGGCGATTTGCTGGGATGAG | GCCGACCCATTTGTTCTCTGC | 57.1 |
| WBGCAS4/5 | TCGCCTAGAGCCCCAAATTCTC | TCATTGTCAGCCTGCCAAGTTG | 58.2 |
| WBGCAS6 | TTGGAGGGCTTCTATGGCTATC | ATGTGTGCGTTTCCAACTTTCC | 55.4 |
| WBGCAS7 | TGTCGCTTTCCAACTGCTACAC | GCTGGGTTCGAGTGGGTGAG | 57.5 |
| WBGCAS8 | ACGCAAATAATGAGCGAAATGC | ACAACTTGTGCAAGCCAACTG | 55.1 |
| WBGCAS9 | GGAAGCAACCAAATGCCATCTG | GTAAATCCGTAAGGGCAACACC | 56.8 |
| WBGCAS10/11 | CCTCTGCAACTCGAAAACCAAG | TGTGCAGGATTACTGTTGTGTC | 56.2 |
| WBGCAS12/13 | TGTGGCTTGAGGCATAAGGAAC | TGTTGCTCTTGCTGCCATGTC | 57.0 |
| WBGCAS14 | GCGACGGTGTGGCATTTTCC | TGGAGCTAGTGCTGGCAGTTG | 58.5 |
| WBGCAS15 | CGACACCACAAGGACCAGAG | TGGAGGTAGCAATGCCTATAGC | 56.0 |
| WBGCAS16 | GGGAGGACGACGATAGTGAGAG | GCGGGTGTTTGGCGTTCAC | 57.5 |
| WBGCAS17 | CGGAGCATTTCCAGAGCCTTG | GCAGCAAAGTCTCGGCGATC | 57.5 |
| WBGCAS18 | CTTGCGTCTGTGTGAAGGTGTG | GCACGCTTACGCTTCCAGTTC | 58.4 |
| WBGCAS19 | AGTGCCTTTGACGGTATATCCC | GTTGCCTTCATTGACCCTTACG | 56.0 |
| WBGCAS20 | AAGAGAGGCCAAGACGAAATCG | ACGTTCTTGCACAAGAGCTTTC | 56.8 |
| WBGCAS21 | GGTCATCGAGCTTAGCAGTTGG | TGTGTGTGATGGGGCAGAGG | 57.5 |
| WBGCAS22 | ACCTCCCACTTCCCTCCATTTC | GGGTGGCTTTGAGAAGGTTGAG | 57.7 |
| WBGCAS23 | GGTCACTGGGCGGGCTAG | TTGCCGTTGTCTGCTTATCACC | 57.2 |
| WBGCAS24/25 | TGACATGTGGCGCTACATATGG | ATCTCCACCTCTAAGCCCAGAC | 56.9 |
| WBGCAS26 | CGGGTCTGTCACGCACTAAC | ATTGGAGGCCAAAGCAAACATC | 56.8 |
| WBGCAS27 | AAGCAGAAGAGGAGGAGGATTC | ACCTCCATTCTTGGGCTTTTCC | 55.3 |
| WBGCAS28 | GGGCAGTGGTTTGGTGAAGC | CCCTCGACCGCTTAGTTCCTC | 57.7 |
| WBGCAS29 | CGTCGTGAGTAACCGCATCATG | TGGCTGGCTGGCACTTACTAC | 58.2 |
| WBGCAS30 | CCATGCACAGTACGTTTGTTTG | ATCCACGTCACTACCGCAATAG | 55.2 |
| WBGCAS31 | ACACAAGAGGCTCGCTGAGAG | AGAGGTACAGAGGTGGGAGGTG | 58.0 |
| WBGCAS32 | CACACAGCCCATCTCCCAATTC | AATCCCACCCACTCGCCATG | 57.6 |
| WBGCAS33/34 | CCAAAATTTCGAACACGGCATG | TGTGTTGACGGGCTCTACCTC | 55.8 |
| WBGCAS35 | TGCACGCTTGGATTGGTTCAG | GTTGTCAGGTGATGGACTTGGC | 57.7 |
| WBGCAS36 | TCCTCAAATTGCCTGCGAGTTG | CACTGACACTGCCAATCACGAG | 57.8 |
| WBGCAS37 | TGCGTCAGCGATGCCTCAG | CTGTCACCACCACCTCTTTTCC | 58.4 |
| WBGCAS38 | CCTCCCTCCCTCACCTCAATAC | TCCGTTCAGGCGAGTGGTAAG | 57.3 |
| WBGCAS39 | CACAGTTGTACGGACGGGAAG | GCTGCCTAATAGGGAGCCTTTG | 56.9 |
| WBGCAS40 | TCTCCATCCCGCACACACTC | TTCTTCGTCGAAAGCCTTGGTC | 57.5 |
| WBGCAS41 | AGCGTGACATCTGGACCTCTG | ACTCTCTCTCCCCTCCCATCC | 57.7 |
| WBGCAS42 | GGCTTGATGCTGGGCTTGG | GCAAAACCCCTCACTCTACTTG | 57.1 |
| WBGCAS43 | TGGGATCTTCTCCTCCAGTCAC | ATCCCATTTGCGCTCTCTCTC | 56.9 |
| WBGCAS44 | GCTTCGGCTGCGATCATAACG | ACCCAAACCTTCCCACCTTCC | 58.4 |
| WBGCAS45 | TCAAGAGCTGCTTCACGTTCAC | TCGGTCGTCGTCTTCAGGTTC | 57.5 |
| WBGCAS46 | TCGGTCGTCGTCTTCAGGTTC | TCAAGAGCTGCTTCACGTTCAC | 58.0 |
| WBGCAS47 | GGGAGGATTCTGACCTGGTACG | ACTTCTTCGGCAACTGTTTGGC | 57.9 |
| WBGCAS48 | CAAGCCTGTTACCTCCGTTCC | AGCGACAGTTGTCAGATTTTCG | 56.9 |
| WBGCAS49 | CGCAAGGATATCATGCCACAAG | CGAAGACTCCGACTTTCCTAGC | 56.2 |
| WBGCAS50 | GGGATCTCTAGCGTTGTGTTGC | CTCCACTTTCCATCGGTGTCTG | 57.7 |
| WBGCAS51 | ACAATGCCTATGGGTCTATCGC | GTGGGGTAAGGCAATCTCTCTC | 56.4 |
| WBGCAS52 | AGCACTCCAGAACTCCGTATCC | GCCACTGCGCTCGGTTTATG | 57.6 |
| WBGCAS53 | AGCTCGACGAACACACCAC | CTCCGCCACTGCACTCAG | 55.8 |
| WBGCAS54 | ACCCAACTTCCCGTCAAACATC | CGCTTTAAGAACGTGGTGTGC | 57.2 |
| WBGCAS55 | GCGTGCGACCCTTCATTAGG | AGGATACAGCCATGTCAGAGAG | 57.2 |
| WBGCAS56 | AAATCCCTTAGGGTTGGCAAAC | AACGTCCACGAAGCATGAAAAC | 55.1 |
| WBGCAS57 | TCCTACGACTCTGAACTTAGCC | ATGCCGTGTGAAAAGTTACCAG | 55.1 |
| WBGCAS58 | ACATGGACACTCTCCCTCTCTC | TGGTTGTGATCCTTAGCTTGCC | 56.7 |
| WBGCAS59 | CCTTTGCCAACCCACAAATTCC | TCCACTGCCCTAATACCACTTG | 56.9 |
| WBGCAS60 | TGGGTTGCCGACTATTTGACAC | CCAACTAACATCTCCGCACACC | 57.2 |
| WBGCAS61 | CACCGCTGACCACCTCTCTC | AGAGTGGTGCTGGATGACGAC | 57.9 |
| WBGCAS62 | GGCGGGACTCTGAAGGGAAC | GTGGGCATTTGAGGCTTAGGTC | 58.1 |
| WBGCAS63 | GGACCATCCAACCCTGCAAG | CATGCTCGCAATGGATCTTGTG | 56.6 |
| WBGCAS64 | GTGTTGTAGTAGGCGGTTTTCC | CCACCATGAATAAGCTCACTGC | 55.7 |
| WBGCAS65 | CCCTTGTCCCTGGCTTCTTC | TGTGTAAGTAGTGCTCGGAGTG | 55.9 |
| WBGCAS66 | AACTGGGGTTCGGATTTCACG | CCCTCACCATCAGACTCCTTTC | 56.8 |
| WBGCAS67 | GGACAACCGCCAGCCAAC | TGTCCCATCTTGGATGCTTTTG | 56.8 |
| WBGCAS68 | GTCTTCGATTCCACTGCTCCTC | CCTCCCTGAACTCCCTCTTCTC | 56.9 |
| WBGCAS69 | ACCAAAGGCGAATTTCAACCAC | GCTCAGCAACGGCAACTACTC | 56.4 |
| WBGCAS70 | CTCCAACGACTAACGGCAACC | TGCCCTTCACAAATTGAACCAC | 57.5 |
| WBGCAS71 | CTCTGACAGTTTGAGGTGTGTG | AGACCCAAACAAACTCACATGC | 55.3 |
| WBGCAS72 | ACTTCAACTTCTTCGGCAACTG | TCCTTTCCTCGTCCGTGTTTTG | 55.5 |
| WBGCAS75 | GGGCTAGGGTTGTCTGGTTTG | GCTGCATCATCTCGGGTGTC | 56.7 |
| WBGCAS76 | ACTTCGGTTTCTGCCACTTGC | TCTTCTAGGCGCTCCACATCTG | 57.6 |
| WBGCAS77 | CTTTGCCATGGTCAAGTTAGGG | CTGGAGACACTATGGTCAACCC | 55.6 |
| WBGCAS78 | TTGTTGAGGAGGTAGCCGAAGG | ACTTCACCACCCACACATACCC | 58.1 |
| WBGCAS79 | CCTTGGCAAATCCTGGCTTCG | GCATCACGTTGGTTGGAGGAC | 58.1 |
| WBGCAS80 | AGAAGGAGGCTGGAAAGAGGAG | CTCAGGGTGCTCAGTTTAGAGG | 57.2 |
| WBGCAS81 | CCTTGGCTTCCTAGTCTGGTG | TTCGGCTCATGGAGACAAAGTG | 55.9 |
