# Supplementary material for: Identification, characterization, and utilization of genome-wide simple sequence repeats to identify a QTL for acidity in apple
Source: BMC Genomics. 2012 Oct 7;13:537. doi: 10.1186/1471-2164-13-537 (PMC3704940; doi:10.1186/1471-2164-13-537)

Additional File 2: Five segregation types of SSRs in the F_1_ mapping population of ‘Jonathan’ x ‘Golden Delicious’


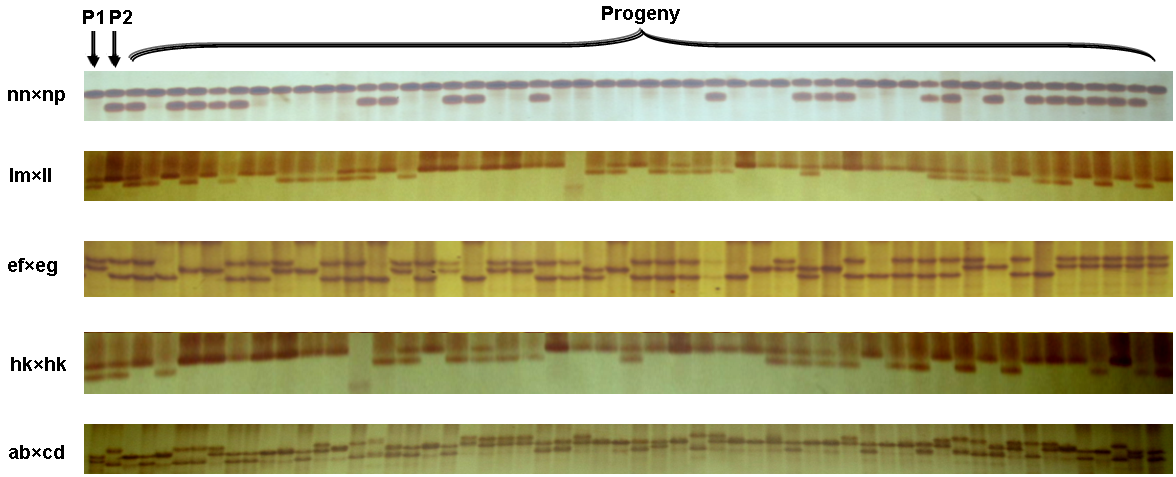

Supplement: Additional file 2 — Five segregation types of SSRs in the F1 mapping population of ‘Jonathan’ x ‘Golden Delicious’. [file 1471-2164-13-537-S2.doc]
