# Supplementary material for: Identification, characterization, and utilization of genome-wide simple sequence repeats to identify a QTL for acidity in apple
Source: BMC Genomics. 2012 Oct 7;13:537. doi: 10.1186/1471-2164-13-537 (PMC3704940; doi:10.1186/1471-2164-13-537)
Supplement: Additional file 4 — Apple SSRs revealing discrepancies between genetic-map and sequence-based physical-map positions. A: Linkage maps of this study, B: Linkage map of previous studies [17,18,31,32], C: The apple draft map. SSRs were anchored onto the draft map of apple by comparing DNA sequences flanking SSRs against the apple genome sequences of cv. ‘Golden Delicious’. [file 1471-2164-13-537-S4.doc]

**Additional File 4: Apple SSRs revealing discrepancies between genetic-map and sequence-based physical-map positions**

| SSR | Linkage group | | Chromosome |
| --- | --- | --- | --- |
|  | A* | B* | C* |
| WBGCAS9 | 14 | N/A | 13 |
| WBGCAS14 | 12 | N/A | 15 |
| WBGCAS27 | 3 | N/A | 6 |
| WBGCAS30 | 5 | N/A | 8 |
| WBGCAS32 | 3 | N/A | 9 |
| WBGCAS39 | 6 | N/A | 12 |
| WBGCAS44 | 6 | N/A | 7 |
| WBGCAS49 | 12 | N/A | 6 |
| WBGCAS61 | 6 | N/A | 11 |
| WBGCAS65 | 10 | N/A | 16 |
| WBGCAS67 | 15 | N/A | 16 |
| WBGCAS78 | 10 | N/A | 5 |
| WBGCAS79 | 15 | N/A | 5 |
| Hi08g06 | 10 | 10 | 17 |
| CH02a10 | 10 | 10 | 3 |
| CH01e09b | 10 | 10 | 15 |
| Hi08g03 | 10 | 10 | 6 |
| Hi23g08 | 4 | 4 | 9 |
| CH04e02 | 4 | 4 | 9 |
| Hi02a03 | 5 | 5 | 9 |
| NZmsEE663789 | 7 | 7 | 12 |
| CH04g12 | 8 | 8 | 7 |
| CH05a02y | 8 | 8 | 15 |
| CH03g06 | 14 | 14 | 11 |
| Hi09f01 | 15 | 15 | 10 |
| Hi11a01 | 15 | 15 | 10 |
| CH04c10 | 17 | 17 | 9 |
| CTG1069981 | 1 | 1 | 3 |
| CTG1076780 | 1 | 1 | 6 |
| CN936557 | 4 | 4 | 17 |
| CTG1061792 | 5 | 5 | 4 |
| CTG1063360 | 10 | 10 | 15 |
| CN855119 | 10 | 10 | 8 |
| CTG1061080 | 10 | 10 | 8 |
| CN868471 | 17 | 17 | 9 |
| BACSSR10 | 11 | 11 | 14 |

A: Linkage maps of this study, B: Linkage map of previous studies [17,18,35,36], C: The apple draft map. SSRs were anchored onto the draft map of apple by comparing DNA sequences flanking SSRs against the apple genome sequences of cv. ‘Golden Delicious’.
